# Use of Hierarchical Carbon Nanofibers Decorated with NiCo Nanoparticles for Highly Sensitive Vortioxetine Determination

**DOI:** 10.3390/ijms232314555

**Published:** 2022-11-22

**Authors:** Joanna Smajdor, Marcel Zambrzycki, Beata Paczosa-Bator, Robert Piech

**Affiliations:** Department of Analytical Chemistry and Biochemistry, Faculty of Materials Science and Ceramics, AGH University of Science and Technology, Al. Mickiewicza, 30-059 Krakow, Poland

**Keywords:** vortioxetine, electrode modifier, voltammetry, carbon nanofibers, nanoparticles

## Abstract

A new voltammetry method for the highly sensitive antidepressant drug vortioxetine (VOR) is presented using glassy carbon electrodes modified with hierarchical carbon nanofibers with NiCo nanoparticles (eCNF/CNT/NiCo-GCE). The electrochemical behavior of VOR was investigated by cyclic voltammetry, which indicates that its oxidation is an adsorption-controlled process with the exchange of two electrons and one proton. The effects of various factors on the VOR peak, such as supporting electrolyte type, preconcentration time, and potential, or influence of interferents, were tested using the square wave voltammetry technique (SWV). The linear voltametric response for the analyte was obtained in the concentration range from 0.01·10^−6^ to 3.0·10^−6^ mol L^−1^ with the detection limit of 1.55·10^−9^ mol L^−1^ for a preconcentration time of 60 s. The proposed method was successfully applied for highly sensitive VOR determination in complex matrices such as tablets, urine, and plasma with good recovery parameter.

## 1. Introduction

Depression is one of the common disorders that affects people around the world nowadays [1]. Therefore, the efforts of researchers are concentrated on the development of new types of drugs that may help patients overcome this disease. Vortioxetine (VOR) (1-[2-(2-dimethyl-phenylsulfanyl)-phenyl]-piperazine hydrobromide) was, for the first time, accepted for the treatment of major depressive disorders in the US in 2013, becoming one of the most commonly prescribed drugs for this disorder in 2017 [2,3,4]. 

Vortioxetine represents a multimodal mechanism of action by influencing the activity of serotonergic receptors and inhibiting the activity of the serotonin transporter [5]. Vortioxetine is a 5-HT_3_, 5-HT7, and 5-HT_1D_ receptor antagonist, partial 5-HT_1B_ receptor antagonist, and 5-HT_1A_ receptor agonist. These features lead to modulation of serotonergic transmission and possibly also noradrenergic, dopaminergic, histamine, acetylcholine, GABA, and glutamate transmission [6,7,8]. The drug has an antidepressant and anxiolytic effect; in animals, it improves cognition, learning, and memory functions [9,10,11].

Several analytical methods have been reported for highly sensitive determination of vortioxetine in different matrices. The most popular high-performance liquid chromatography (HPLC) method coupled with different detectors allows one to detect not only VOR, but also its active metabolites in human body fluids [12,13,14,15,16,17,18]. However, despite the high sensitivity and selectivity of the measurements, they required expensive instrumentation and lots of environmentally unfavorable organic solvents. Therefore, voltametric methods may be an alternative approach for VOR determination, considering their ease, low cost, and high sensitivity of measurements. In the literature different types of working electrode, such as carbon-nanotube-modified stencil printed electrode [19], graphite pencil electrode [20], glassy carbon electrode modified with gold nanoparticles and graphene [21], or with three-dimensional nickel ferrite nanospheres [22] were already used for vortioxetine determination.

In this work, electrochemical studies on vortioxetine are presented. The VOR behavior was investigated using glassy carbon electrodes modified with hierarchical electrospun carbon nanofibers with NiCo nanoparticles (eCNF/CNT/NiCo-GCE), and a square wave voltammetry technique was developed for VOR high sensitivity determination. A new procedure was successfully applied for its sensitive determination in pharmaceutical formulations, synthetic urine, and plasma samples.

## 2. Results and Discussion 

### 2.1. Characterization of Nanocomposite

**Figure 1:**
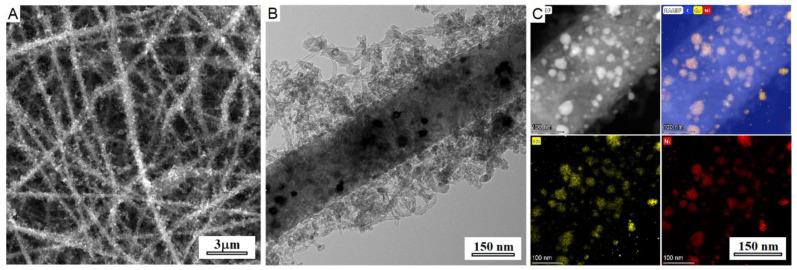
(**A**) SEM and (**B**) TEM images of eCNF/CNT/NiCo nanocomposite. (**C**) HAADF-STEM/EDX mapping showing elemental distribution in the NiCo nanoparticles.

The SEM and TEM images of eCNF/CNT/NiCo hierarchical nanocomposite are shown in Figure 1. The observations confirmed successful formation of metallic nanoparticles, and growth of carbon nanoprotrusions on the surface of carbon nanofibers. The metal nanoparticles were located mostly on the surface of nanofibers, which evidences prevailing base-growth of carbon nanotubes. The elemental mapping revealed homogenous distribution of metals in nanoparticles and did not show signs of segregation, proving the alloy character of the NiCo phase. The mean diameters of electrospun nanofibers, carbon nanotubes, and NiCo nanoparticles were 245.4, 17.0, and 18.4 nm, respectively.

**Figure 2:**
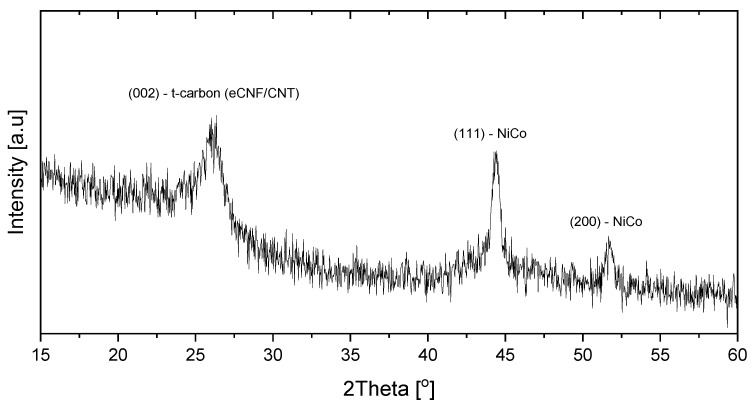
X-ray diffraction pattern of eCNF/CNT/NiCo nanocomposite.

The XRD diffraction pattern of the nanocomposite is shown in Figure 2. The presence of two diffraction peaks at 2θ~44.45° (111) and 51.67° (200) confirms successful reduction of metal oxides and formation of fcc-NiCo phase. The wide feature at 26.35° comprises two diffraction peaks coming from the (002) planes of eCNF and CNT phases, whereas the CNT component should be attributed to higher 2θ. At the position of about 43° the increased background can be noted which indicates the presence of (100) graphitic planes. The shift of (002) feature towards lower angular positions and lack of the reflexes from the other (hkl) planes demonstrates the turbostratic nature of carbon [23]. More detailed information regarding the synthesis and structure of eCNF/CNT/NiCo nanocomposites can be found in our previous works [24,25].

### 2.2. Voltammetric Characterization 

**Figure 3:**
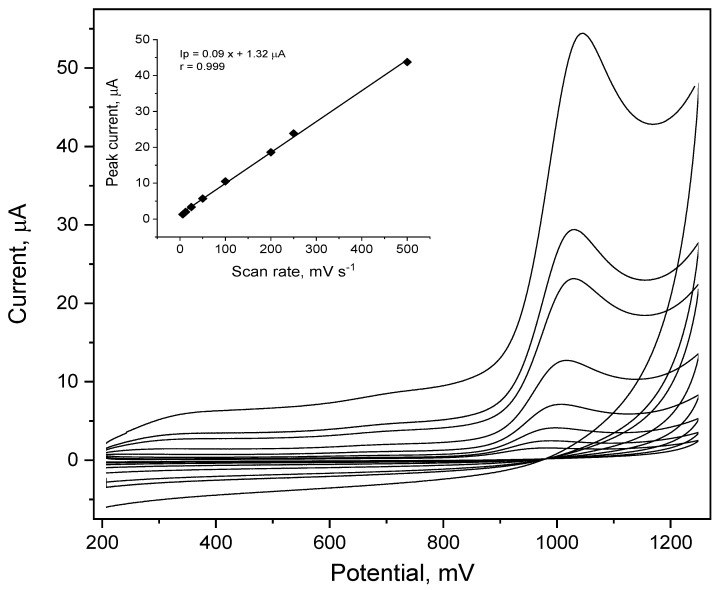
Cyclic voltammograms of the 5 µmol L^−1^ of vortioxetine registered in the 0.1 mol L^−1^ phosphate buffer (pH 6.0) for the scan rate values of: 6.3; 12.6; 25; 50; 100; 250, and 500 mV s^−1^, and the linear dependence of the VOR peak on the scan rate value.

The vortioxetine behavior on the eCNF/CNT/NiCo-GC electrode was investigated using the linear sweep voltammetry technique. Measurements were made in 0.1 mol L^−1^ phosphate buffer (pH 6.0) and the VOR concentration was equal to 5 µM. CV voltammograms were registered in the potential range from 200 to 1250 mV with scan rates in the range of 6.3–500 mV s^−1^. The results of the measurements are presented in Figure 3. In each scan a well-shaped anodic peak is visible with no presence of the cathodic peak, indicating that the VOR oxidation performed on the eCNF/CNT/NiCo-GC electrode is an irreversible process. It may be observed that the VOR peak potential value was shifting toward the more positive values with the increasing scan rate value. To investigate the nature of the oxidation process, the plot between the vortioxetine peak current versus the scan rate and the square root of the scan rate was crossed out. Considering the linear dependence (Equation (1)) obtained for the relationship between peak current vs. scan rate, it is possible to say that the VOR oxidation process is adsorption controlled (Figure 3).

(1) $$I_{p}=0.09x+1.32\;\mu A,r=0.999$$

**Figure 4:**
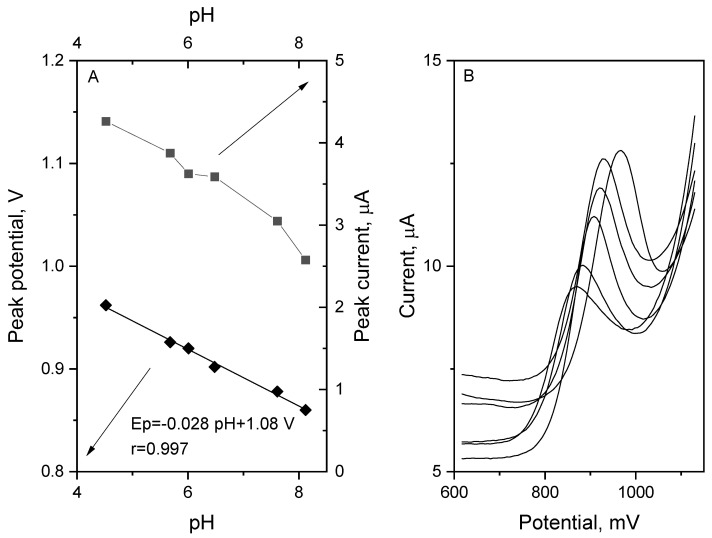
Dependence of the VOR peak current and potential on pH (**A**) and corresponding voltammograms (**B**) measured for the pH values from the range 4.5–8.1 for 1 µmol L^−1^ of VOR in 0.1 mol L^−1^ phosphate buffer. Other parameters are as in point 2.7.

To obtain the high stability and repeatability of the vortioxetine peak, the 0.1 mol L^−1^ phosphate buffer with a pH of 6.0 was chosen. Both the VOR peak current and the peak potential were strongly influenced by the supporting electrolyte pH value. Therefore, the dependence of these parameters in the function of changing pH was crossed out and is presented in Figure 4. The range of pH values tested in the experiment was from 4.5 to 8.1. The VOR peak current was characterized by the highest values with pH of 4.5, but the stability and repeatability of the signal, especially for the low concentration of VOR, was weak. Therefore, for quantitative vortioxetine measurements, the phosphate buffer with a pH value of 6.0 was chosen. While the pH value of the supporting electrolyte was changing into higher values, the VOR peak potential was shifting toward less positive values. The dependence between the VOR peak current and electrolyte pH may be expressed in the following Equation (2).

(2) $$E_{p}=-0.028\;pH+1.08\;V$$

The slope value obtained—0.028 V pH^−1^—suggests that the number of electrons exchanged in the vortioxetine oxidation process doubles the protons. The number of electrons exchange in the oxidation reaction may be calculated using the following Equation (3):

(3) αn=0.048|Ep−Ep12|

**Scheme 1:**
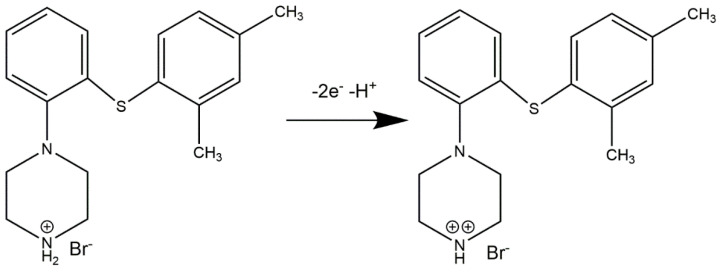
Proposed mechanism of vortioxetine electrochemical oxidation.

Considering obtained data, the mechanism of possible vortioxetine oxidation reaction is presented in Scheme 1. 

### 2.3. Influence of the SWV Parameters on Vortioxetine Peak

The choice of optimal parameters for the SWV technique is crucial to obtain high sensitivity of the vortioxetine signal on eCNF/CNT/NiCo-GC electrode. The following parameters were investigated in a wide range of values: pulse potential (dE), step potential (Es), frequency (f), waiting, and sampling time (t_p_ and t_w_). The best results were obtained with the following values: dE = 50 mV, Es = 6 mV, f = 50 Hz, and t_p_ = t_w_ = 5 ms; therefore, these values were chosen for all measurements performed.

### 2.4. Influence of the Supporting Electrolyte on the Vortioxetine Peak

The choice of the proper supporting electrolyte for high-sensitivity measurement is one of the crucial points during method optimization. The choice of the supporting electrolyte was optimized considering the possibility of achieving the best sensitivity of the vortioxetine determination. Thus, during the process of choosing the optimal environment, the measurements of different types of supporting electrolytes were investigated. Not only the type of electrolyte was different, but the pH was also a crucial parameter in the choice. The main criteria to meet was to obtain well shaped vortioxetine peak with low-capacity current value. Therefore, the following solutions were examined: 0.05 mol L^−1^ acetate buffer with pH 5.5, 4.5, and 3.8; 0.01 mol L^−1^ borate buffer pH 9.2; 0.1 mol L^−1^ ammonia buffer pH 8.2; 0.1 mol L^−1^ phosphate buffer saline (PBS) pH 7.4; 0.1 mol L^−1^ Britton–Robinson buffer pH 7.0; 0.1 mol L^−1^ dipotassium phosphate pH 9.1; 0.1 mol L^−1^ dihydrogen phosphate pH 4.6; and 0.1 mol L^−1^ phosphate buffer pH 6.0 (Figure not included). When comparing the obtained voltammograms, the best VOR signals considering the peak shape, peak current value, and relation of the peak to the base line were registered in 0.1 mol L^−1^ phosphate buffer; therefore, it was chosen for other experiments.

### 2.5. Influence of Preconcentration Time and Potential on Vortioxetine Peak

**Figure 5:**
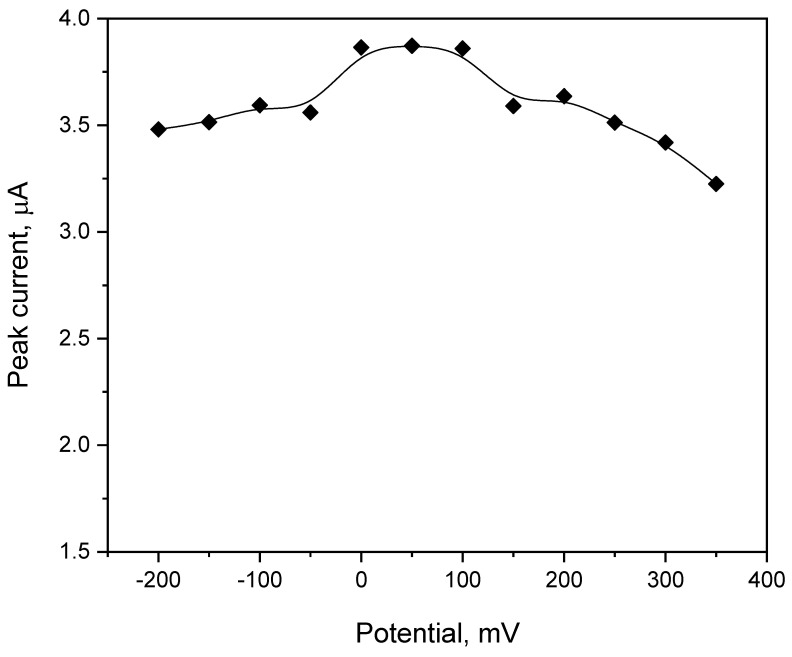
The influence of the preconcentration potential on VOR peak current value. Other instrumental parameters as in point 2.7.

The effect of the preconcentration potential and time on the value of the vortioxetine peak current was examined in a wide range. The preconcentration potential was studied from −200 to 350 mV with a change of the 50 mV. The value of the VOR peak current achieved the lowest figure for the preconcentration potential of -150 mV (3.22 µA), whereas the highest peak current value was registered for 0 mV (3.87 µA). The full dependence of the VOR peak current value on the preconcentration potential value is presented in Figure 5. Taking into account the results obtained, the preconcentration potential of 0 mV was chosen for further measurements.

**Figure 6:**
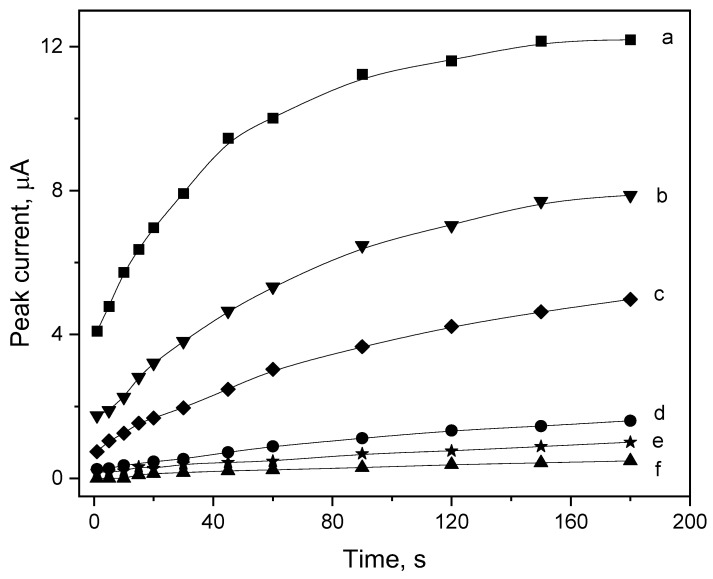
The influence of the preconcentration time on VOR signal for its concentration of (a) 1 µmol L^−1^; (b) 0.5 µmol L^−1^; (c) 0.25 µmol L^−1^; (d) 0.1 µmol L^−1^; (e) 0.05 µmol L^−1^ and (f) 0.01 µmol L^−1^. Other instrumental parameters as in point 2.7.

The dependence of the vortioxetine peak current on the preconcentration time, measured for six different VOR concentrations, is presented in Figure 6. For each concentration, the vortioxetine oxidation peak current value was increasing in size, and after reaching the maximum, the signal became stable. The maximum height for the VOR concentration of 1 µmol L^−1^ was 12.2 µA (t_acc_ = 180 s), while for 0.01 µmol L^−1^ it was equal to 0.4871 µA (t_acc_ = 180 s).

### 2.6. Interferences

Organic and non-organic interferents may affect the analyte signal in a few ways, i.e., by reduction/oxidation in the same potential as the analyte, which may strengthen the signal, or by adsorption to the electrode surface and in consequence suppressing the analyte electrochemical signal by blocking the active centrums to which the analyte may connect. The use of modifiers may influence the effects of interferents on the analyte peak by changing their reduction or oxidation potential, which may work both ways: by the separating or imposing of the analyte and interferent peaks, and in the end it may result in their better differentiation, or prevent the quantitative analysis. Under optimized conditions, the influence of potential organic and inorganic interferents on the VOR peak current was investigated. The results showed that the additions of the following substances, such as Zn (II), Al (III), Pb (II), Mo (IV) (2 µmol L^−1^), Mg (II), Ca (II), Na (I) (1 mmol L^−1^), Cu(II) (20 µmol L^−1^), NH_4_^−^, SO_4_^2+^, NO_3_^−^ (1 mmol L^−1^), and CO_3_^2−^ (0.2 mmol L^−1^) did not cause a difference in the VOR peak current value of VOR. However, the difference in the VOR signal was observed for the addition of 0.2 mmol of L^−1^ Cl^−^ (a decrease of 14%), 2 µmol L^−1^ Fe (III) (a decrease of 18%), and 2 µmol L^−1^ Mn (II) (an increase of 16%). Moreover, the addition of organic compounds that may possibly occur in pharmaceutical samples or body fluids was tested. The addition of glucose (0.1 mmol L^−1^), lactose monohydrate (60 µmol L^−1^), ascorbic acid, citric acid (0.2 mmol L^−1^), caffeine, aspartame, microcrystalline cellulose, and magnesium stearate (20 µmol L^−1^) did not interfere with the vortioxetine peak.

### 2.7. Analytical Performance

**Figure 7:**
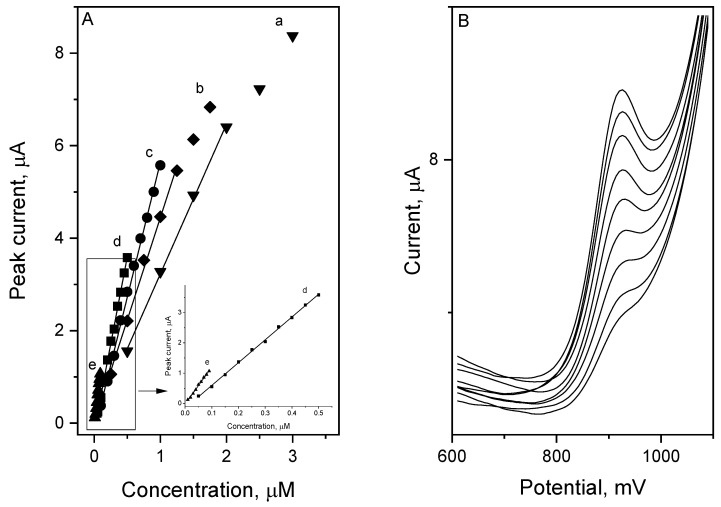
SWV vortioxetine calibration curves obtained for preconcentration times of (a) 5 s; (b) 10 s; (c) 15 s; (d) 20 s, and (e) 60 s (**A**), and corresponding voltammograms for the preconcentration time of 60 s (**B**). Other instrumental parameters as in point 2.7.

The SWV vortioxetine voltammograms in the range of concentration from 0.01·10^−6^ to 3.0·10^−6^ mol L^−1^ and preconcentration times of 5–60 s are presented in Figure 7. The VOR detection limit was calculated from the dependence between signal noise (*s*) and slope of the calibration curve (*b*) (Equation (4)).

(4) $$LOD=\frac{3.3s}{b}$$

Taking this into account, the detection limit of vortioxetine for the preconcentration time of 60 s was calculated as 1.55·10^−9^ mol L^−1^. The LOD value was compared with VOR detection limits obtained by different analytical methods and is presented in Table 1.

The repeatability of the sensor was also measured and expressed as RSD for seven consecutive measurements of 0.25 µmol L^−1^ VOR on eCNF/CNT/NiCo-GC electrode and was equal to 2.3%.

**Figure 8:**
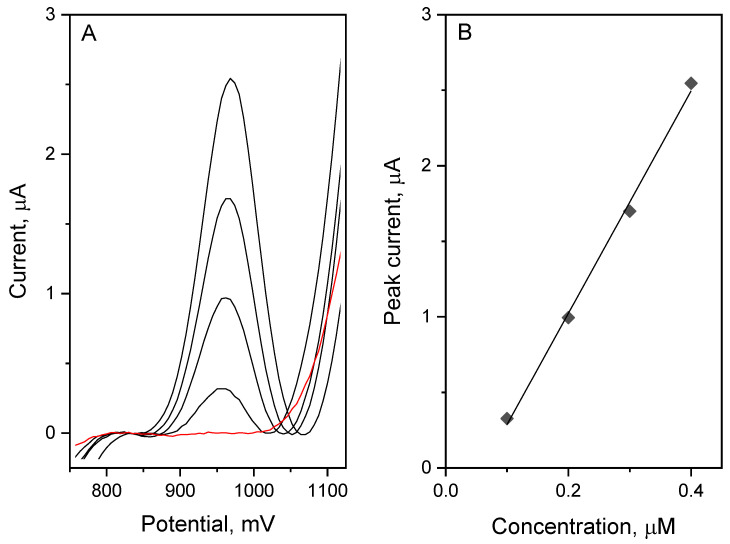
SWV vortioxetine voltammograms registered in plasma sample after background correction (plasma background voltammogram marked as red) (**A**) with corresponding calibration curve (**B**). Other instrumental parameters as in point 2.7.

To validate the proposed determination method, vortioxetine was measured in tablet, urine, and plasma samples. All measurements were performed using the standard addition method, using three additions of the synthetic VOR standard of known concentration. The sample preparation is presented in points 2.3–2.5. In the measurements, the urine sample was diluted 5× and the plasma sample was diluted 20×. The results are presented in the Table 2 and the obtained voltammograms with the corresponding plot are presented in Figure 8. The recovery parameter was very good, calculated in the range from 95.0 to 102.8%. Therefore, the analytical usefulness of the proposed method for highly sensitive determination of vortioxetine in real samples using the glassy carbon electrode modified with hierarchical nanocomposite—carbon nanofibers/carbon nanotubes/NiCo nanoparticles—was confirmed.

## 3. Materials and Methods

### 3.1. Measuring Apparatus

A M161 multipurpose electrochemical analyzer with the M164 electrode stand (mtm-anko, Warsaw, Poland) was used in all electrochemical measurements. The EAQt software was used to register and analyze the obtained voltammograms, while Origin Lab 2021b software was used for quantitative analysis and visualization of the data. The three-electrode quartz voltametric cell with a volume of 20 mL was used in all voltametric measurements. As a working electrode, glassy carbon electrode modified with the hierarchical nanocomposite—electrospun carbon nanofibers/carbon nanotubes/NiCo nanoparticles (eCNF/CNT/NiCo-GCe)—as the reference electrode Ag/AgCl/KCl (3 mol L^−1^) was used, and as an auxiliary electrode a platinum wire was used. During all measurements, stirring of the electrolyte was performed using a magnetic Teflon-coated bar. pH measurements were performed using the Elmetron CX-705 (Zabrze, Poland). The sonication of the prepared solutions was carried out using an ultrasonic bath (Intersonic IS-1K, Olsztyn, Poland). The eCNF/CNT/NiCo nanocomposite was obtained according to a combined synthesis procedure involving electrospinning of precursor nanofibers with organometallic complexes of Ni and Co, and subsequent heat treatment coupled with the synthesis of carbon nanotubes from the gas phase. Detailed description of the synthesis procedure can be found in [23]. The GCE-modifier suspension was prepared by sonicating the as-obtained nanocomposite in dimethylformamide (1 mg ml^−1^) for 5 min, as according to the methodology described in our previous work [26].

### 3.2. Characterization of Nanocomposite

The observations of morphology of nanocomposites were performed using a Quanta 650 FEG scanning electron microscope (SEM) working with a standard secondary electron detector. The used acceleration voltage was from 10 to 15 kV. The high-resolution imaging was carried out using an FEI Tecnai TF20 X-TWIN (FEG) transmission electron microscope (TEM) at the acceleration voltage of 200 kV. Energy-dispersive X-ray spectroscopy (EDS) was performed with an EDAX RTEM 0.3 sr, HAADF accessory coupled with the microscope for elemental mapping. The X-ray diffraction patterns were collected using a PANalytical X-Pert Pro X-ray diffractometer equipped with copper Cu Kα radiation source (λ = 1.546 Å). The measurements were performed in grazing incidence mode, in 2θ range from 15 to 60°.

### 3.3. Chemicals

The standard solution of vortioxetine hydrobromide (5 mL, 0.01 mol L^−1^) was obtained by dissolving an appropriate amount of this reagent (TRC, Toronto, ON, Canada) in equal proportions of water and ethanol (POCH) and then stored in the fridge. Phosphate buffer (1 mol L^−1^, pH 6.0) was prepared by mixing the proper amounts of KH_2_PO_4_ and K_2_HPO_4_ (both Merck). Lyophilizate urine (Medidrug Basis-line U) was purchased from Medichem, and human plasma was purchased from Biowest. All reagents were of analytical grade and used without further purification. All solutions were prepared with double-distilled water. For the synthesis of nanocomposites, polyacrylonitrile (M_W_ = 150,000 Da) from MilliporeSigma, and cobalt and nickel acetylacetonates from ACROS Organics, were used. Nitrogen, hydrogen, and acetylene used during thermal treatment were provided by AIR LIQUIDE. Dimethylformamide (Avantor Performance Materials Poland S.A) was used as solvent for electrospinning and for preparation of modifier inks.

### 3.4. Tablet Sample Preparation

For VOR measurements of VOR in the pharmaceutical formulation, Brintellix tablets (H. Lundbeck) (10 mg of VOR or 12.71 mg VOR-HBr per tablet) were purchased from the local drugstore. For the analysis of the vortioxetine content in the tablet formulation, three of them were crushed in a mortar and then quantitatively transferred to the volumetric flask and dissolved in ethanol and water (proportions 1:1) and sonicated for 5 min. Afterwards, homogenization of the obtained solution was filtrated using syringe cellulose filters (pore size 0.45 µm) (Biosens, Rio Grande do Sul, Brazil) and used in further measurements.

### 3.5. Urine Sample Preparation

For VOR measurements of VOR in the urine, a freeze-dried urine sample was purchased from Medidrug. The content of the vial was dissolved according to the manufacturer’s manual, in 5 mL of double-distilled water. After complete dissolution, the sample was filtrated using a syringe filter with a pore size of 0.45 µm (Biosens). This prepared sample was used in further measurements.

### 3.6. Plasma Sample Preparation

For the measurements of VOR in the plasma, an appropriate plasma sample was purchased from Biowest. According to the manufacturer’s manual, the sample was stored in a freezer, at −20 °C, and defrosted directly before measurements. To remove protein components present in the sample, 800 µL of plasma was mixed with 200 µL of 10% trichloroacetic acid (TCA). The mixture was shaken for 2 min on the vortex and then centrifuged for 30 min with an approximate speed of 10,000 rpm. The obtained supernatant was then filtrated using a syringe filter with a pore size of 0.45 µm (Biosens). This obtained sample was used in further measurements.

### 3.7. Working Electrode Preparation

Before modification of the electrode surface, a glassy carbon electrode with diameter size of 3 mm was polished using alumina slurry with particle size of 0.3 µm (Buechler). After being rinsed under a strong stream of double distilled water, the GC electrode was placed in the beaker with methanol and then placed in the ultrasonic bath for 3 min. When the electrode surface was dry, 5 µL of the eCNF/CNT/NiCo (DMF) modifier suspension was dropped onto the glassy carbon surface and left to evaporate the solvent (approximately 3 h). Before placing the modifier, its mixture was stirred in the ultrasonic bath for 45 min to obtain a fully homogeneous solution. After this preparation, the electrode was ready for measurements.

### 3.8. Measurement Procedure

For quantitative measurements of vortioxetine, the square wave voltammetry (SWV) technique was applied. Measurements were made in the 0.1 mol L^−1^ phosphate buffer (pH 6.0). Before the first curve registration, the electrode was stabilized by performing approximately 20 measurements using the cyclic voltammetry technique. The instrumental parameter of the SWV was as follows: sampling and waiting time t_p_ = t_w_ = 5 ms, step potential E_s_ = 6 mV, pulse amplitude dE = 50 mV, and frequency f = 50 Hz. The voltammograms were registered in the potential range of 200–1150 mV, after the preconcentration step described by the parameters: E_acc_ = 0 mV and t_acc_ = 5 s. Between the measurements, a rest period of 20 s was strictly required in order to obtain the high repeatability of the VOR signal.

## 4. Conclusions

In this work a new, highly sensitive determination method of vortioxetine determination was proposed using a glassy carbon electrode modified with hierarchical nanocomposites: electrospun carbon nanofibers/carbon nanotubes/NiCo nanoparticles. The proposed procedure performed using the square wave voltammetry technique allows one to achieve a VOR detection limit of 1.55 nmol L^−1^ (preconcentration time of 60 s). The reproducibility of the method measured and expressed as RSV is very good, and for the VOR concentration of 0.25 µmol L^−1^ is equal to 2.3%. The presented procedure was applied for vortioxetine determination in real samples—tablets, synthetic urine, and plasma—with very good recovery parameters (95.0–102.8%), which implies the usefulness of this method for highly sensitive VOR determination in such samples. All of the above leads to the conclusion that the described method is suitable for fast, simple, and accurate determination of vortioxetine using glassy carbon electrodes modified with hierarchical nanocomposite—carbon nanofibers/carbon nanotubes/NiCo nanoparticles.

## Figures and Tables

**Table 1:** Comparison of the vortioxetine detection limits obtained using other analytical methods.

Electrode	LOD	Reference
CNTs-SP	1·10^−8^ mol L^−1^	[19]
graphite pencil electrode	2·10^−8^ mol L^−1^	[20]
AuNPs@GRP/GCE	50·10^−9^ mol L^−1^	[21]
3D NiFe2O4 NS/AGNP/GCE	0.55·10^−9^ mol L^−1^	[22]
eCNF/CNT/NiCo-GCE	1.55·10^−9^ mol L^−1^	This work

CNTs-SP—carbon nanotube bulk modified stencil printed electrode; AuNPs@GRP/GCE—gold nanoparticles/graphene nanocomposite modified glassy carbon electrode; 3D NiFe_2_O_4_ NS/AGNP/GCE—glassy carbon electrode with three-dimensional nickel ferrite nanospheres-modified activated graphite nanoplatelets; eCNF/CNT/NiCo-GCE—hierarchical nanocomposite—carbon nanofibers/carbon nanotubes/NiCo nanoparticles.

**Table 2:** Results of vortioxetine determination in samples: tablets, urine, and plasma.

Sample	Added, µM	Found, µM	Recovery, %
Tablet	0	0.21 ± 0.01	-
	0.10	0.32 ± 0.03	95.0
	0.20	0.41 ± 0.03	99.7
	0.30	0.51 ± 0.01	98.9
Urine (5× diluted)	0	ND	-
	0.25	0.24 ± 0.03	102.8
	0.50	0.51 ± 0.04	98.6
	0.75	0.76 ± 0.01	98.8
	1	0.99 ± 0.02	100.7
Plasma (20× diluted)	0	ND	-
	0.10	0.10 ± 0.03	101.3
	0.20	0.20 ± 0.01	98.8
	0.30	0.30 ± 0.02	100.3
	0.40	0.40 ± 0.01	100.0

ND—not detected.

## Data Availability

The data presented in this study are available on request from the corresponding author.
